# Practical Feasibility of Digital Cognitive Assessments in Older Kenyan Adults

**DOI:** 10.1002/alz70857_105987

**Published:** 2025-12-25

**Authors:** Kevin Kipkoech, Wambui Karanja, Cynthia Isabel Smith, Rachel W Maina, Jasmit Shah, Elena Tsoy, Sarah McDonagh, Shireen Javandel, Victor Valcour, Chinedu Udeh‐Momoh, Karen Blackmon

**Affiliations:** ^1^ Brain and Mind Institute, Aga Khan University, Nairobi, Nairobi, Kenya; ^2^ SNF Global Center for Child and Adolescent Mental Health, Child Mind Institute, New York, NY, USA; ^3^ Brain and Mind Institute, Aga Khan University, Nairobi, Kenya; ^4^ Memory and Aging Center, University of California San Francisco, San Francisco, CA, USA; ^5^ Global Brain Health Institute, University of California, San Francisco, San Francisco, CA, USA; ^6^ Wake Forest University, School of Medicine, Winston‐Salem, NC, USA; ^7^ Geisel School of Medicine at Dartmouth, Hanover, NH, USA

## Abstract

**Background:**

Digital cognitive assessments have emerged as promising tools in limited resource settings, with the potential for adaptability and scalability in Alzheimer's Disease and Related Dementia research. However, it is important to demonstrate their practical usability relative to traditional paper‐based tests for widespread adoption. This study compares the feasibility of both modalities in a healthy older adult multilingual Kenyan population.

**Method:**

We enrolled 135 community‐dwelling Kenyan adults [mean (SD) age in years=55.2 (8.6), min = 45, max=79; 64.4% female], ranging in education from primary to doctoral‐level [median education = 11 years]. Participants completed a cognitive test battery that included the Tablet‐based Cognitive Assessment Tool [TabCAT] and paper‐based cognitive tests assessing memory, attention, and executive function. All tests were available in English and culturally adapted to Swahili. Feasibility metrics included error types, language‐switching (as a potential indicator of increased cognitive load), valid task completion, learning curves (as a reflection of user engagement), and practice trial success.

**Result:**

Digital assessments had a 12.9% error rate, mainly due to technical issues (10.6%); whereas, paper‐based tests had an 8.2% error rate, mainly due to examinee‐related issues (4.2%). Language‐switching was common in paper‐based tests, particularly in tasks involving months (88%) and numbers (97%) but was less common in digital tests, ranging from 5% to 12%. Completion rates were high for digital tasks such as Birdwatch (100%), Match (100%), Line Orientation (100%), and Flanker (98%), with slightly lower rates for Set‐Shifting (90%). Completion rates were 100% for paper‐based tests, except for Trail making Test A (3% of participants timed out). Positive learning curves were apparent across paper‐based and digital learning trials on memory tests, indicating effective user engagement. Digital test practice trials had high success rates prior to actual test trials (100% for Flanker and Match, 97% for Set‐Shifting, 87% for Line Orientation), indicating effective task design and user engagement.

**Conclusion:**

Culturally and linguistically adapted digital tools have the potential for scalable, user‐friendly, and adaptable cognitive testing in resource‐limited settings. Technical issues with digital tests and the additional cognitive load of language‐switching on working memory tasks should be addressed prior to scaling.

